# In Situ Reconstruction of NiCo–MOF Enhances Acetate Production Through Ethanol Electro‐Oxidation in Alkaline Medium

**DOI:** 10.1002/smsc.70248

**Published:** 2026-04-10

**Authors:** Yash Kumar Yadav, Anshika Sahu, Biplab Kumar Manna, Anirudha Shekhawat, Rajib Samanta, Sudip Barman

**Affiliations:** ^1^ School of Chemical Sciences National Institute of Science Education and Research (NISER) Bhubaneswar Orissa India; ^2^ Homi Bhabha National Institute Mumbai Maharashtra India

**Keywords:** acetic acid, ethanol electrooxidation, faradaic efficiency, in situ reconstruction, metal–organic framework

## Abstract

Electrochemical ethanol oxidation (EOR) is promising strategy for value‐added chemical production, such as acetic acid, while replacing energy intensive oxygen evolution reaction at the anode. However, developing efficient, affordable electrocatalysts is crucial for practical ethanol electrolysis to enhance the EOR kinetics, reduce overpotential, and minimize energy losses. In this context, we synthesized a nickel‐cobalt‐based metal‐organic framework (NiCo–MOF) as a precatalyst for ethanol oxidation. The NiCo oxyhydroxides were formed by the in situ reconstruction of the MOF during the cycling process. This reconstructed composite exhibits outstanding electrocatalytic performance toward EOR, achieving a current density of 10 mA/cm^2^ at 1.33 V (RHE). The reconstructed NiCo–MOF showed a maximum FE of ∼95.6% for acetate production at 50 mA/cm^2^ current density. The better catalytic activity of reconstructed NiCo–MOF compared to other samples is due to its strong synergistic interaction between Ni and Co, which facilitates the preoxidation, thereby increasing the EOR. The catalyst also possesses a stable ethanol electrolysis through 50 h. The overall ethanol electrolysis on the catalyst needs 220 mv less potential compared to water electrolysis at 10 mA/cm^2^ current density. The result suggests that in situ reconstructed bimetallic MOF could be an efficient electrocatalyst for alcohol electrooxidation for potential green energy integration.

## Graphical abstract

NiCo–MOF was synthesized as a precatalyst and electrochemically reconstructed into active NiCo oxyhydroxides during cycling. The reconstructed catalyst shows superior EOR activity, requiring only 1.33 V vs. RHE to reach 10 mA cm^−2^ and shows ∼ 95.6% Faradaic efficiency for acetate at 50 mA cm. This demonstrates reconstructed bimetallic MOFs as efficient, selective, and stable catalysts for alcohol electrooxidation and green energy integration.

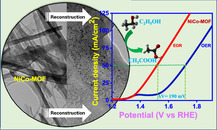

## 1 Introduction

One of the most pressing challenges of the digital age is the depletion of fossil fuel supplies and the harmful environmental effects of exploiting nonrenewable resources due to our growing energy needs, which are driven by our growing energy needs fueled by a rapidly expanding population [1, 2, 3, 4]. Renewable energy sources such as solar, tidal, and wave power are good substitutes for supplying energy needs, but their intrinsic intermittency limits their use due to seasonal and regional fluctuations [5, 6]. Hydrogen can be considered as a future fuel [7]. This is due to its higher energy density, environmental friendliness, and zero carbon dioxide emissions with just water as a byproduct [8, 9, 10]. The most sustainable method for H_2_ production at present is electrocatalytic water electrolysis [11]. Water splitting consists of two distinct half‐reactions: the hydrogen evolution reaction (HER) occurring at the cathode (2H^+^ + 2e^−^ → H_2_) and the oxygen evolution reaction (OER) taking place at the anode (4OH^−^ → 2H_2_O + O_2_ + 4e^−^) [12, 13]. The theoretical voltage for water electrolysis is 1.23 V, but the slow kinetics of the OER reduce overall efficiency [14]. This kinetic limitation significantly hampers the overall efficiency of the water‐splitting process for hydrogen production [15, 16, 17]. To overcome this bottleneck, replacing the anodic OER with the oxidation of small organic molecules has emerged as a promising strategy to reduce cell voltage while simultaneously producing value‐added chemicals [18, 19, 20]. Numerous studies have explored the anodic oxidation of small organic molecules such as methanol, ethanol, ethylene glycol, glycerol, and benzyl alcohol [21, 22, 23, 24, 25, 26]. In contrast to the oxygen evolution reaction (OER), these processes usually require much lower cell voltages, allowing for the simultaneous creation of hydrogen at the cathode and the production of valuable compounds at the anode.

The ethanol oxidation reaction (EOR) offers several advantages in terms of sustainability and energy efficiency compared to other alcohol‐based fuels. Ethanol has played an important role as both an energy carrier and a mechanistic probe in investigations of organic oxidation reactions [27]. Ethanol can be efficiently produced using renewable feedstocks through two primary methods: the hydrolysis of lignocellulosic biomass followed by microbial fermentation, or the direct fermentation of sugar‐rich crops [28]. Unlike methanol and other conventional fuels, ethanol is non‐toxic, renewable, and environmentally friendly. Additionally, it possesses a high specific energy of 8.01 kWh kg^−1^, comparable to gasoline, making it a desirable and sustainable energy source [29]. Consequently, direct ethanol fuel cells (DEFCs) have attracted significant research attention due to the numerous advantages of ethanol, including its high volumetric energy density, broad availability from renewable biomass sources, and ease of storage and transportation [30, 31]. Despite these benefits, the sluggish kinetics of the anodic EOR pose a significant obstacle to the commercialization of DEFCs [29]. The EOR mechanism is widely accepted to proceed via dual pathways, the C_1_ and C_2_ routes. Since ethanol's carbon–carbon (C—C) bonds store most of its chemical energy, effective C—C bond breakage via the C_1_ pathway is necessary to maximize energy extraction [32]. However, in most practical systems, ethanol undergoes partial oxidation through the C_2_ route, resulting in partial oxidation products and diminished fuel utilization efficiency [33, 34]. Additionally, carbonaceous intermediates, such as adsorbed carbonyl (CO_ads_) species from the C_1_ pathway and acetyl (CH_3_CO_ads_) species from the C_2_ route, can strongly adsorb onto and poison active catalytic sites, significantly reducing electrocatalytic performance. Therefore, improving the stability and efficiency of DEFCs requires a rational catalyst design that encourages C—C bond cleavage while avoiding intermediate poisoning [35].

The reaction kinetics of ethanol electrooxidation can be significantly enhanced by developing and utilizing more efficient electrocatalysts. Platinum and other platinum‐group metals (PGMs) are widely acknowledged as benchmark electrocatalysts due to their high activity; however, their high cost and susceptibility to CO poisoning, limit their practicality in large‐scale applications. These limitations have driven research efforts toward alternative, cost‐effective materials, particularly in alkaline media, where a broader range of catalyst compositions can be employed. To address these challenges, numerous research groups have developed a variety of mono‐, binary‐, ternary‐, and quaternary‐metal‐based electrocatalysts incorporating both noble and non‐noble metals for anodic ethanol electrooxidation [36]. Accordingly, there is growing interest in developing non‐noble, structurally tunable electrocatalysts that can simultaneously enhance activity and stability.

Metal–organic frameworks (MOFs) have recently garnered considerable attention as emerging electrocatalysts, owing to their highly ordered porosity, exceptional structural tunability, and diverse morphologies [37, 38, 39]. MOFs can function either as direct electrocatalysts or serve as precursors for deriving active catalytic materials [40, 41, 42]. Their intrinsic features, such as high surface area, adjustable pore size, and flexible composition, make them particularly attractive for energy‐related electrochemical applications [43, 44, 45, 46, 47]. Among these, transition metal‐based MOFs stand out as promising low‐cost candidates for catalysis due to their rich redox chemistry and the ease of tailoring their structure at the molecular level. In this context, bimetallic MOFs (BMOFs), which incorporate two different metal ions with similar electronic configurations and charge densities, have emerged as particularly appealing [48, 49, 50]. These frameworks often exhibit enhanced electrocatalytic activity compared to their monometallic counterparts, attributed to synergistic effects between the two metal centers. The improved performance of BMOFs is often linked to their modulated electronic structure, such as favorable valence states and optimized e_g_ orbital occupancy, which enhances charge transfer and catalytic activity. Additionally, the interplay between structural flexibility, defect engineering, and electronic disorder in these hybrid materials opens new avenues for fine‐tuning their electrocatalytic behavior [15]. Among the non‐noble metals, nickel (Ni) has emerged as a highly promising candidate due to its favorable electronic properties, cost‐effectiveness, and proven activity in a range of energy‐related applications. Cobalt (Co), often paired with nickel in bimetallic systems, further enhances electrocatalytic performance owing to its compatible atomic radius, stable oxidation states, and synergistic interaction with Ni, which promotes the preoxidation of the catalyst and increases the catalytic activity [51, 52]. Wang et al. synthesize CoNi‐PHNs for EOR, which shows 1.39 V vs RHE potential [53]. Cabot and coworkers established a solution‐based method to prepare Ni_1‐*x*_Co_*x*_Se_2_ NPs with different metal ratios for EOR, exhibits 1.34 V vs RHE potential [54]. Brunner groups reports synthesis of Cu_1.0_ Zn_0_@C MOF having 1.37V vs RHE [55].

In this context, a bimetallic NiCo–MOF was used as a precatalyst for the ethanol oxidation. The MOF was reconstructed after 15 cycles, and the reconstructed material was used as a catalyst for ethanol oxidation. Incorporating multiple metal centers enables the preoxidation of the material, which improves the charge transfer and the formation of abundant active sites, thereby increasing the catalytic activity. The catalyst also possesses a maximum faradic efficiency of 96.5% at 50 mA/cm^2^ current density for acetate production from ethanol. This bimetallic reconstructed MOF also exhibits excellent overall ethanol electrolysis performance when combined with commercial Pt/C at the cathode in a two electrode system.

## 2 Experimental Section

### 2.1 Preparation of NiCo–MOF

**SCHEME 1:**
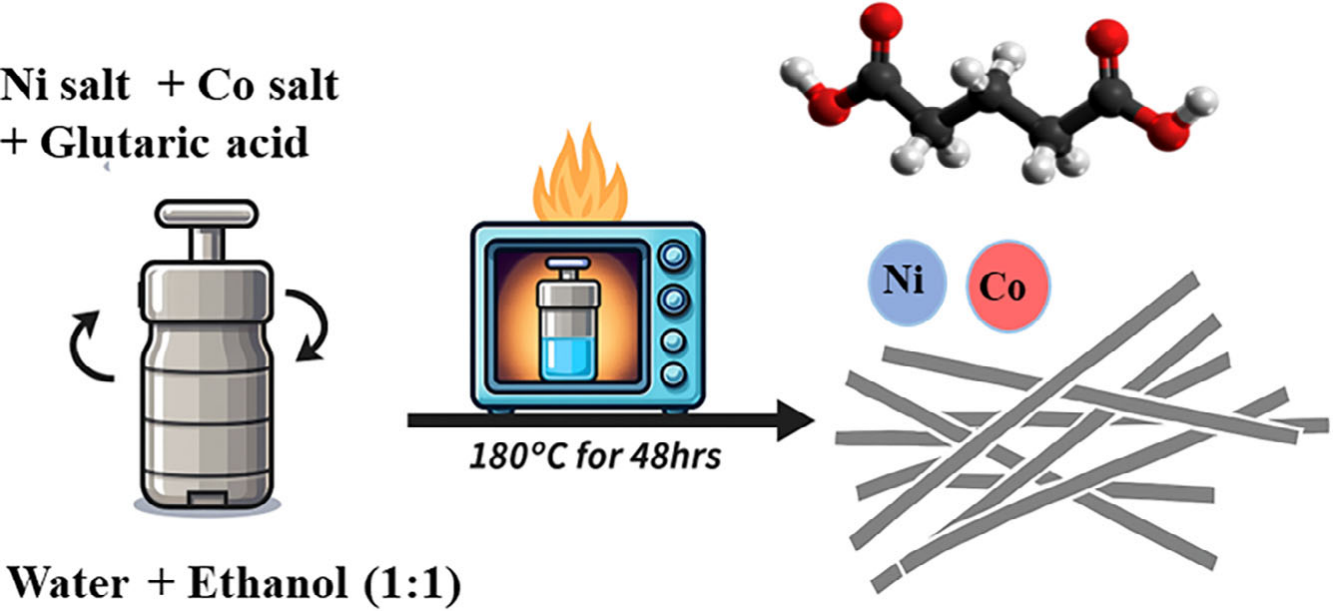
Schematic synthesis procedure of the NiCo–MOF.

NiCo–MOF was synthesized via a modified hydrothermal method based on our previously reported procedure by Panda et al. [56]. In a typical synthesis method, 0.79 g of glutaric acid, 0.64 g of potassium hydroxide, nickel acetate tetrahydrate, and cobalt(II) nitrate tetrahydrate were dissolved in a 1:1 solution of deionized water and ethanol (40 mL). To this solution, 4 mL of 0.4 M NaOH was added gradually while stirring continuously to achieve a homogeneous mixture. Here, NaOH and KOH were employed as alkaline modulators to control solution pH and promote deprotonation of glutaric acid, which is essential for regulated metal–ligand coordination and MOF framework formation. The gradual addition of NaOH enabled controlled nucleation and homogeneous Ni^2+^/Co^2+^ incorporation, while KOH contributed to the overall alkaline environment and ionic strength during solvothermal growth. The homogeneous precursor solution was carefully transferred into a 100 mL Teflon‐lined stainless‐steel autoclave and subjected to solvothermal treatment at 180°C for 48 h. Upon completion of the reaction, the resulting solid was obtained, thoroughly washed several times with deionized water and ethanol to flush out residual precursors and impurities, and subsequently desiccated under vacuum. The obtained product was named as NiCo–MOF. A systematic approach to the synthesis of NiCo–MOF is illustrated in Scheme 1.

In addition, Ni–MOF and Co–MOF were synthesized using the same synthesis method using only Ni(CH_3_COO)_2_·4H_2_O and Co(NO_3_)_2_·4H_2_O, respectively.

## 3 Results and Discussions

### 3.1 Structural and Morphological Characterizations

**FIGURE 1:**
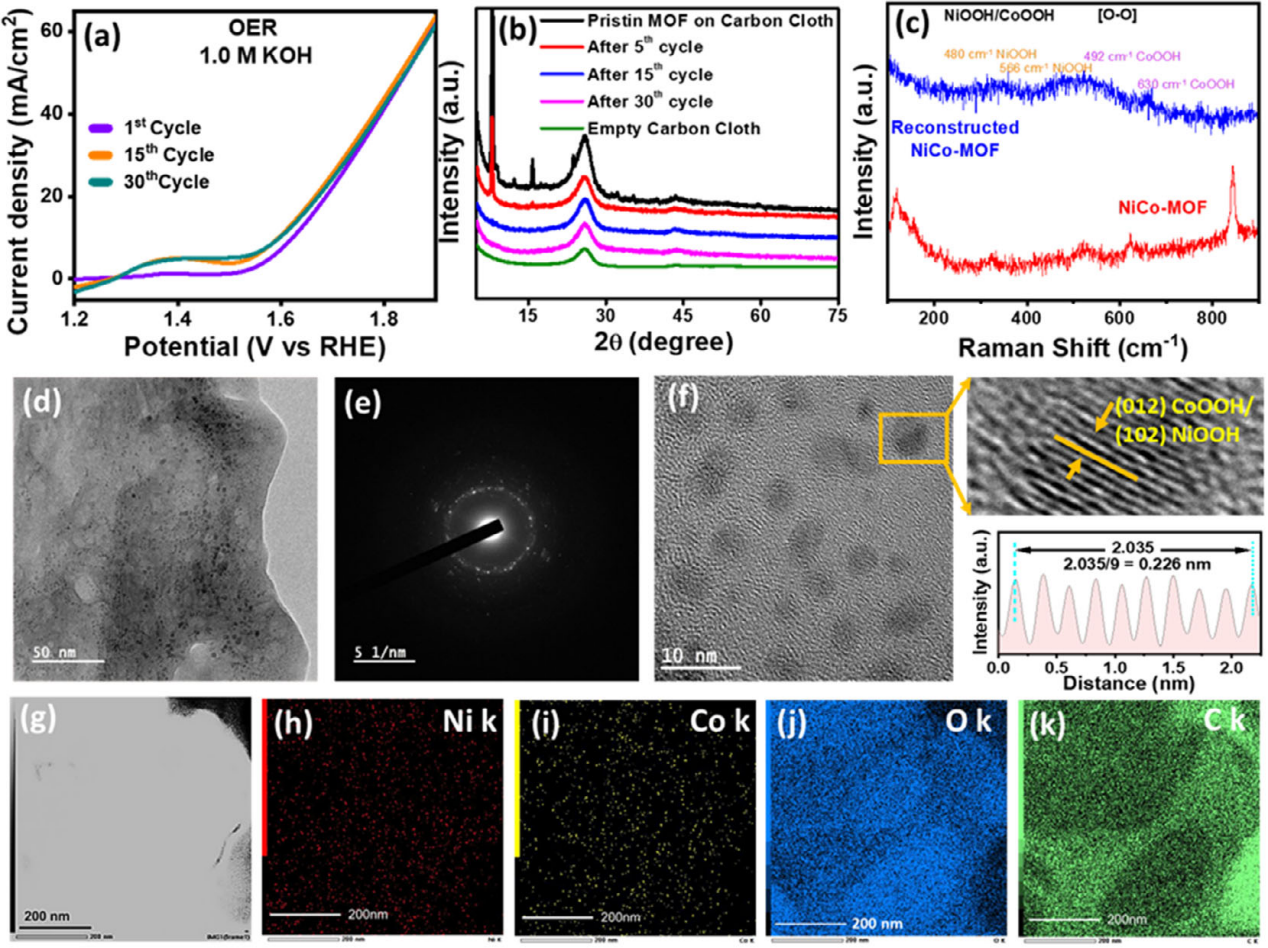
(a) LSV curves of NiCo–MOF up to 30 cycles. (b) p‐XRD pattern of catalysts after different cycles. (c) Raman spectra of the reconstructed catalysts. (d–f) TEM, SAED, and HRTEM image of the reconstructed catalyst, respectively. (g–k) STEM‐EDS elemental mapping of the reconstructed catalyst.

The NiCo–MOF was synthesized following the previously reported procedure [56]. To confirm the morphology and structure, field‐emission scanning electron microscopy (FESEM), transmission electron microscopy (TEM), and powder X‐ray diffraction (p‐XRD) were performed. The synthesized MOFs’ phase purity and crystallographic structure were analyzed using p‐XRD. It was found that the p‐XRD pattern in Figure S1a well matched the reported MOF [54], confirming the formation of NiCo–MOF. Figure S1b shows the FESEM image of NiCo–MOF, and Figure S1c–f show TEM images of the NiCo–MOF and monometallic Co & Ni MOF, respectively, showing one‐dimensional (1D) nanobelt‐like morphology, and the SAED image shows the polycrystalline nature of the MOF. The MOF was used as a precatalyst for electrochemical ethanol oxidation. Generally, the MOFs are electrochemically unstable. During electrochemical performance, the structure collapses, and the material phases are reconstructed. We also observed the reconstruction of the NiCo–MOF. We tracked the reconstruction process using p‐XRD, Raman, and TEM analysis. We performed 30 cycles of linear sweep voltammetry (LSV) in the potential range of 1.1–1.9 V vs. RHE, as shown in Figure 1a. To track structural reconstruction via p‐XRD, shown in Figure 1b, the characteristic peaks of the pristine MOF completely disappeared after 15 cycles, indicating that the structural transformation of the MOF was completed within the initial 15 cycles.

However, no information about the catalyst structure or the reconstructed material from the p‐XRD pattern was drawn. Thus, for more information on the material structure, phases, element composition, and morphology, we performed Raman, TEM, and X‐ray photo‐electron spectroscopy (XPS) analysis of the reconstructed material. Figure 1c shows the Raman peaks of pristine MOF and catalyst after 15 cycles; some peaks were found at different positions, at 480 and 566 cm^−1^ and at 492 and 630 cm^−1^, which could be due to the formation of NiOOH and CoOOH respectively [57, 58]. The TEM images further suggest the change of the morphology from nanobelts to small nanoparticles after the 15 cycles shown in Figure 1d. The SAED image reveals the catalyst's polycrystalline structure. The presence of small bright spots within the circles suggests the presence of crystalline phases in the compound, as shown in Figure 1e. Figure 1f shows the HRTEM image of the reconstructed catalyst, calculating lattice fringes reveals crystalline planes, and the d‐spacing value of 0.226 is related to the (012) and (102) planes of CoOOH and NiOOH, respectively. Elemental mapping was also performed to determine the elemental compositions of the reconstructed catalyst and NiCo–MOF, as shown in Figures 1g–k and S2a–d, respectively. The elemental analysis confirms the presence of nickel (Ni), cobalt (Co), carbon (C), and oxygen (O). STEM‐EDS elemental mapping shows composition, visualization of elemental distribution, interfaces, and material heterogeneity in complex structures.

**FIGURE 2:**
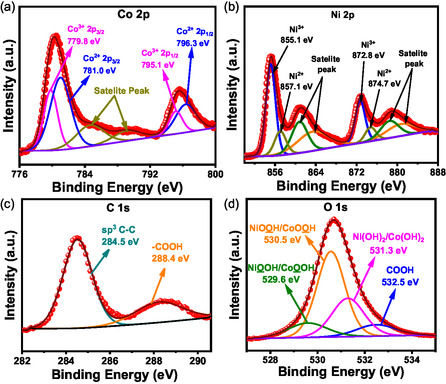
(a–d) High‐resolution XPS spectra of Co 2p, Ni 2p, C 1s, and O 1s, respectively.

Figure S2a–d show elemental mapping of NiCo–MOF. STEM‐EDS spectra provides quantitative metal loading data, visualized through percentage distribution charts across the sample's microstructure (Figure S3a). Table S1 shows the total metal loading chart of MOFs. For the NiCo–MOF, the Ni and Co ratio is nearly 1:1. Further, X‐ray photoelectron spectroscopy (XPS) was carried out to determine the oxidation states of the elements in the reconstructed catalyst. Figure S3b gives information about the metal percentages present in our reconstructed catalyst, determined from the XPS analysis. Figure S3c shows the XPS survey scan of the reconstructed catalyst, which suggests the presence of Ni, Co, C, and O in the material. Figure 2a shows a high‐resolution Co 2p XPS spectrum of the reconstructed catalyst. The spectrum was fitted with six peaks. The peak positioned at 781.0 eV (2p_3/2_) and 796.3 eV (2p_1/2_) corresponds to Co^2+^, and the peak positioned at 779.8 eV (2p_3/2_) and 795.1 eV (2p_1/2_) is attributed to Co^3+^ [59]. Additional peaks observed in the XPS spectrum correspond to Co 2p satellite features, which are characteristic indicators of cobalt oxide phases. This supports the presence of CoOOH/Co_3_O_4_ species in the reconstructed NiCo–MOF composite. Figure 2b shows a high‐resolution Ni 2p XPS spectrum of the reconstructed catalyst. The spectrum was fitted with eight peaks. The peaks at 855.1 eV (2p_3/2_) and 872.8 (2p_1/2_) eV correspond to Ni^3+^, and the peak positioned at 857.1 eV (2p_3/2_) and 874.7 eV (2p_1/2_) is attributed to Ni^2+^, suggesting the presence of NiOOH in the reconstructed catalyst. Other peaks are due to the satellite peak of Ni 2p [58, 60]. The fitted C 1s XPS spectrum reveals four distinct peaks (Figure 2c). The peaks at binding energies of 284.5 eV and 288.4 eV are attributed to sp^3^‐hybridized carbon and C—C bonding, as well as the carboxyl (–COOH) functional group, respectively. Figure 2d shows the O 1s XPS spectrum, which was fitted using four peaks. The peak at 531.3 eV indicates the presence of –OH due to NiOOH–CoOOH/Ni(OH)_2_ –Co(OH)_2_, while the peaks at 529.6 eV suggest the oxygen Ni–O present in NiOOH/CoOOH, and 532.5 eV are due to the O present in carboxylic (–COOH) functional group [58, 59]. The above experimental data suggests that during the cycling process the pristine MOF structure degrades and forms NiOOH and CoOOH active species, which further takes part in the reactions.

## 4 Electrochemical Analysis

### 4.1 Electrochemical Ethanol Oxidation Reaction (eEOR)

**FIGURE 3:**
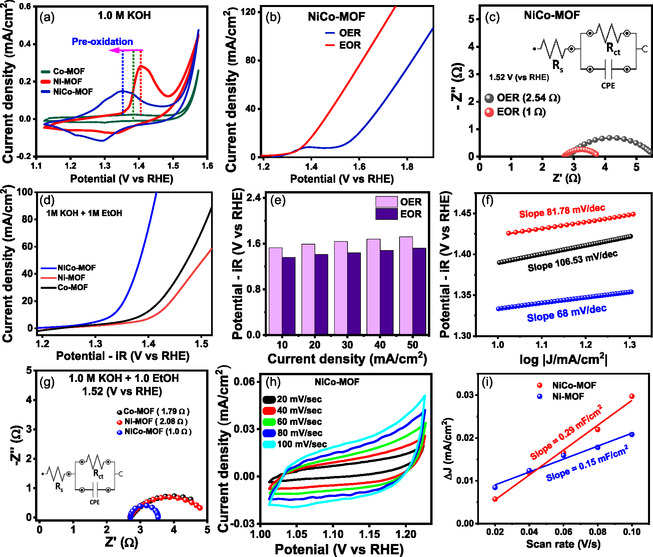
(a) Cyclic voltammetry plots of NiCo–MOF, Co–MOF, and Ni–MOF. (b,c) Non‐iR‐corrected OER and EOR polarization curves and corresponding Nyquist plots of NiCo–MOF. (d) the EOR polarization plots of all MOFs. (e) Comparison of potentials at various current densities for NiCo–MOF. (f,g) Tafel and Nyquist plots of all MOFs. (h) CVs of NiCo–MOF at different scan rates. (i) Δ*j* vs scan rate plots of NiCo–MOF.

The electrochemical oxygen evolution reaction (OER) performance of all catalysts was evaluated using a standard three‐electrode setup, employing Ag/AgCl as the reference electrode, platinum wire as the counter electrode, and catalyst‐loaded carbon cloth as the working electrode. All measurements were conducted with the same metal loading across the samples to ensure consistency. The cyclic voltammetry (CV) curve of reconstructed NiCo–MOF, Co–MOF, and Ni–MOF in 1 M KOH, as shown in Figure 3a, exhibits characteristic oxidation peaks at 1.38 and 1.40 V (vs RHE), corresponding to the redox couple of Co^2+^/Co^3+^ and Ni^2+^/Ni^3+^, respectively. Comparatively, the NiCo–MOF displays Co^2+^ preoxidation at 1.352 V (vs RHE), which is relatively low compared to Co–MOF and Ni–MOF, indicating that the combination of Ni^2+^ and Co^2+^ in NiCo–MOF promotes the preoxidation of Co^2+^ or Ni^2+^, accompanied by OH* production, which is favorable for further electrocatalysis. To determine the optimal catalyst loading for peak activity, OER LSV measurements were performed with increasing loading, as shown in Figure S4. It shows that 3 mg MOF loading is optimal and is used for further measurements. Figure S4b shows the OER activities of all the MOFs. After the addition of ethanol, the anodic current density increases gradually, and the corresponding redox couples between Co and Ni disappear, suggesting the active involvement of Co and Ni in the EOR (Figure 3b).

It was found that the required potential to reach a current density of 10 mA/cm^2^ for OER and EOR is 1.53 and 1.35 V, respectively, which is significantly lower for the EOR. The electrochemical impedance spectra of NiCo–MOF in the presence and absence of ethanol at 1.52 V are shown in Figure 3c. This shows a smaller charge transfer resistance (R_ct_ = 1 Ω) in the presence of ethanol, suggesting that the EOR is kinetically more favorable than the OER. The electrochemical activity of all composites was carried out in the presence of 1 M ethanol in 1 M KOH, Figures 3d and S5 shows the iR‐corrected and Non‐iR‐corrected ethanol oxidation polarization curve for all the MOFs. NiCo–MOF and monometallic Co and Ni MOF require 1.33, 1.39, and 1.41 V to reach 10 mA cm^−2^ current density. The ethanol oxidation reaction (EOR) requires lower potentials compared to the oxygen evolution reaction (OER) in all current densities, as illustrated in Figure 3e. The superior EOR performance of NiCo–MOF is further confirmed by its lower Tafel slope of 68 mV/dec, compared to 81.78 mV/dec for Ni–MOF and 106.53 mV/dec for Co–MOF, indicating significantly faster ethanol oxidation kinetics for NiCo–MOF (Figure 3f). EIS analysis reveals that a lower charge transfer resistance (R_ct_) facilitates faster ethanol oxidation reaction (EOR) kinetics due to enhanced electron transfer. The Nyquist plot (Figure 3g) shows that NiCo–MOF exhibits the smallest semicircle, indicating its significantly lower R_ct_ compared to monometallic MOFs, and thus superior catalytic activity. A comparison table of NiCo–MOF with other reported catalysts is shown in Table S2. The cyclic voltammetry measurements of the composites at various scan rates were carried out within the non‐faradaic region to determine the double‐layer capacitance (C_dl_) (Figures 3h and S6a, b). The electrochemical surface area (ECSA) correlates directly with the C_dl_. The double‐layer capacitance of NiCo–MOF, having a Co and Ni ratio of 1:1, is 0.29 mF/cm^2^, which is notably higher than that of Ni–MOF, which is 0.15 mF/cm^2^. This suggests a larger electrochemically active area and a greater abundance of active sites in NiCo–MOF (Figure 3i). Hence, these findings indicates that both Ni and Co in the NiCo–MOF are essential for enhanced catalytic activity in electrochemical ethanol oxidation.

**FIGURE 4:**
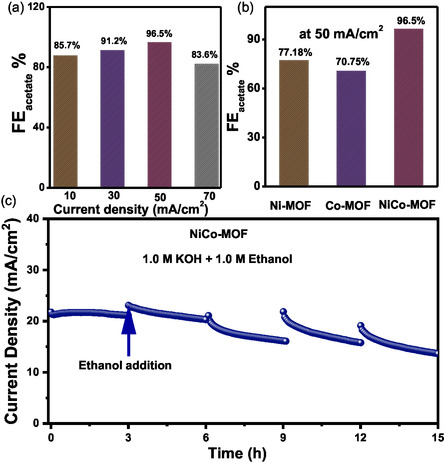
(a) FE plot at different current densities for NiCo–MOF. (b) FE plot of all MOFs at 50 mA/cm^2^. (c) Long‐term stability plot of NiCo–MOF during the EOR.

The electrochemical EOR was investigated under ambient pressure and temperature. For product analysis, chronopotentiometry was performed for 0.5 h for all catalysts at different current densities. In a customized H‐type electrochemical cell, all experiments were conducted using 15 mL of an electrolyte composed of 1.0 M ethanol in 1.0 M KOH as the anolyte, while the catholyte compartment contained 15 mL of 1.0 M KOH solution. The anode and cathode chambers were separated by a Nafion 117 proton‐exchange membrane to prevent cross‐contamination of reaction products. Following chronopotentiometric measurements, the liquid‐phase products generated from ethanol oxidation were collected from the anolyte and characterized using ^1^H NMR. The faradaic efficiency (FE) was calculated at different current densities (10, 30, 50, and 70 mA cm^−2^) for NiCo–MOF composites with varying the Co and Ni ratio (Figure 4a). The highest FE of 96.5% was achieved at 50 mA/cm^2^ with NiCo–MOF. In comparison, monometallic Ni–MOF and Co–MOF exhibited significantly lower FEs of 77.18% and 70.75%, respectively, under the same current density (Figure 4b).

Figure S7 shows the NMR spectrum of the reaction mixture after electrolysis, revealing the formation of acetic acid as an oxidative product of ethanol. The long‐term stability of NiCo–MOF was also evaluated at 1.5 V for 15 h with the addition of 1.0 M ethanol at a 3 h time interval (Figure 4c). The current density was found to increase sharply close to its initial point with adding ethanol each time. This suggests good stability of the catalyst over a long time.

Therefore, the experimental results demonstrate that preoxidation induced by synergistic interactions among the composite components is a key factor leading to the boosted catalytic performance. This conclusion is further supported by comparative electrochemical analyses of the physical mixture and other control samples (Figure S8). Moreover, the reduced charge–transfer resistance (Rct) and increased electrochemically active surface area (ECSA) play pivotal roles in delivering superior electrocatalytic activity toward the EOR.

### 4.2 Overall Electrolysis Performance of Coupling the EOR with the HER

**FIGURE 5:**
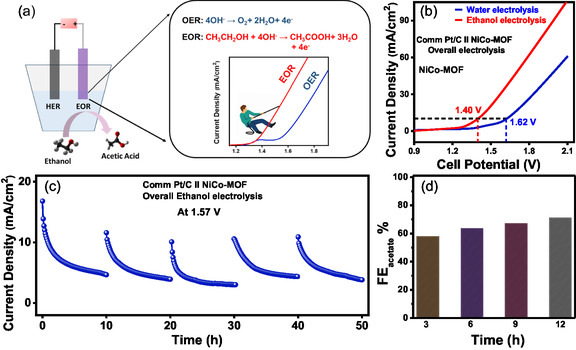
(a) Schematic diagram for overall ethanol electrolysis and water splitting. (b) LSV curves of overall ethanol electrolysis and water splitting using Pt/C||NiCo–MOF. (c) 50 h stability plot at a constant voltage of 1.57 V; (d) FE plot at 3, 6, 9, and 12 h for the overall ethanol electrolysis system.

To assess electrocatalytic activity under more realistic conditions, a conventional electrolyzer (two‐electrode system) was set up and operated at room temperature, where NiCo–MOF and commercial Pt/C act as an anode (EOR) and cathode (HER), respectively. Figure 5a illustrates the layout of the two‐electrode setup employed for overall ethanol electrolysis. The catalyst demonstrates excellent efficiency for overall ethanol electrolysis, requiring only a cell potential of 1.40 V to achieve a current density of 10 mA/cm^2^. In contrast, water electrolysis with the same setup demands a higher cell potential of 1.62 V to reach the same current density (Figure 5b). To evaluate the stability of the NiCo–MOF catalyst for overall ethanol electrolysis, chronoamperometry was performed at a constant voltage of 1.57 V over 50 h (Figure 5c).

Faradaic efficiency (FE) measurements taken at four successive time intervals confirmed sustained catalytic performance, underscoring reconstructed NiCo–MOF as a promising and stable catalyst for ethanol electrolysis applications (Figure 5d).

## 5 Conclusions

In summary, reconstructed NiCo–MOF exhibits an excellent EOR performance in alkaline solution, which shows a low potential of 1.33 V vs RHE to achieve the current density of 10 mA cm^−2^, along with high faradic efficiency and selectivity for ethanol to acetic acid conversion. The high EOR activity of reconstructed NiCo–MOF is attributed due to its synergistic interaction between Ni and Co, high ECSA, and active area. In addition, for the overall ethanol electrolysis, NiCo–MOF needs 1.40 V to reach 10 mA cm^−2^ current density. This study opens a route to the potential of utilizing readily available transition metal‐based MOFs for electrocatalytic ethanol oxidation, promoting the advancement of sustainable chemistry and renewable energy technologies, and thereby bolstering the environmental and economic sustainability of ethanol‐utilizing industrial processes.

## Supporting Information

Additional supporting information can be found online in the Supporting Information section. Materials and instruments; electrochemical experiments; ECSA, TOF, and Tafel slope calculations; additional characterization and EOR plots; EOR activity comparison.

## Conflicts of Interest

The authors declare no conflicts of interest.

## Supporting information

Supplementary Material

## Data Availability

The data that support the findings of this study are available from the corresponding author upon reasonable request.
