# Supplementary material for: In Situ Reconstruction of NiCo–MOF Enhances Acetate Production Through Ethanol Electro‐Oxidation in Alkaline Medium
Source: Small Sci. 2026 Apr 10;6(4):e70248. doi: 10.1002/smsc.70248 (PMC13073222; doi:10.1002/smsc.70248)
Supplement: Supplementary file 1 — Supplementary Material [file SMSC-6-e70248-s001.pdf]

In-situ Reconstruction of NiCo-MOF Enhances Acetate Production through Ethanol Electro-oxidation in Alkaline Medium

Yash Kumar Yadav,[a,b] Anshika Sahu,[a,b] Biplab Kumar Manna,[a,b] Anirudha Shekhawat,[a,b] Rajib Samanta[a,b] and Sudip Barman*[a,b]

[a] School of Chemical Sciences, National Institute of Science Education and Research (NISER), Bhubaneswar, Orissa-752050, India.

[b] Homi Bhabha National Institute, Training School Complex, Anushakti Nagar, Mumbai – 400094.

*S.B.: E-mail: sbarman@niser.ac.in; Tel: +91(674)2494183.

Materials:

Nickel (II) acetate tetrahydrate [$Ni(CH_3)_2 \cdot 4H_2O$] and Glutaric acid ($HOOC(CH_2)_3COOH$) was purchased from Sigma Aldrich. Cobalt (II) nitrate tetrahydrate ($Co(NO_3)_2 \cdot 4H_2O$) was purchased from CDH. Ethanol was bought from Thermo Scientific (India). Sodium hydroxide (NaOH) and Potassium hydroxide (KOH) were purchased from Merck. Fisher Scientific provided deuterium oxide (D_2O). The Nafion 117 proton exchange membrane and Toray carbon cloth (TGP-H-60) were bought from Sigma-Aldrich and Alfa Aesar, respectively. All these chemicals were used without any additional purification. Mili-Q water was obtained from an ultra-filtration system (Mili-Q, Millipore) with a measured conductivity of 35 mho cm^{-1} (resistivity of 17.9 MΩ cm) at 25 ^oC.

Physicochemical characterization:

Surface morphology analysis was carried out using a field-emission scanning electron microscope (Carl Zeiss, Germany, Model ∑igma). For FE-SEM imaging, samples were prepared by drop-casting 10 µL of a 1 mg/mL solution onto a silicon wafer and then dried at 45 °C. Powder X-ray diffraction (p-XRD) patterns were obtained using a Bruker DAVINCI D8 ADVANCE diffractometer equipped with Cu K_α radiation (λ = 0.15406 nm). High-resolution transmission

electron microscopy (HRTEM; JEOL F200, operating at 200 kV) was used for detailed surface morphology analysis of the synthesized materials. For TEM imaging, 10 µL of an ethanolic solution (1 mg/mL) was drop-cast onto a TEM grid and air-dried at approximately 45 °C. X-ray photoelectron spectroscopy (XPS) measurements were performed using a VG Microtech instrument, with samples drop-cast onto a silicon wafer. A monochromatic Mg K_{α} X-ray source was used for excitation. Raman spectroscopy was performed using a LabRAM HR system (Horiba) with a 525 nm laser. The pH of the working solution was measured using a Hanna HI 2209 pH meter at room temperature (298 K). Ultrasonic bath sonication was carried out using equipment from Genei Laboratories Private Limited, Bangalore, India. Proton nuclear magnetic resonance (^1H NMR) spectra were recorded using a Bruker 400 MHz NMR spectrometer employing the water suppression method.

Electrochemical characterization:

All the electrochemical experiments were performed in a three-electrode glass cell system, and experimental data were collected through an electrochemical workstation (Autolab, Metrohm, PGSTAT 320N). Electrochemical analysis of NiCo-MOF was carried out by evaporating 1 mL of water and ethanol suspension of NiCo-MOF (3 mg/mL) on a carbon cloth electrode with 1.0 cm^2 surface area as a working electrode; for the counter electrode, a platinum wire electrode was used, and for the reference electrode, Ag/AgCl in 3 M KCl was used. For the conversion of potential to RHE from Ag/AgCl, the Nernst equation was used. $E_{RHE} = E_{Ag/AgCl} + E^0_{Ag/AgCl} + 0.059$ pH, where $E_{Ag/AgCl}$ is the working potential, $E^0_{Ag/AgCl} = 0.1976$ at 250 °C. The current density reported herein was normalized by the geometrical area of the working electrode, and all potentials were reported in the reversible hydrogen electrode (RHE) scale. Oxygen flow was maintained over the electrolyte

(1.0 M KOH) during electrochemical measurements of the oxygen evolution reaction to ensure the O_2/H_2O equilibrium at 1.23 V vs. RHE. Scanning of the working electrodes was performed several times until the curves were stabilized. Then, polarization curves were collected and reported after correction for the iR contribution within the cell. The polarization curves for OER and EOR were recorded at a scan rate of 10 mV/s. To prepare the catalyst-modified carbon cloth electrode in detail, 3 mg of NiCo-MOF (4.99 wt% Ni & 4.59 wt% Co) was dispersed in 1 mL of water using ultrasonication for 10 min. Then, all of the catalyst suspension was drop-cast onto the carbon cloth electrode and dried under a lamp source.

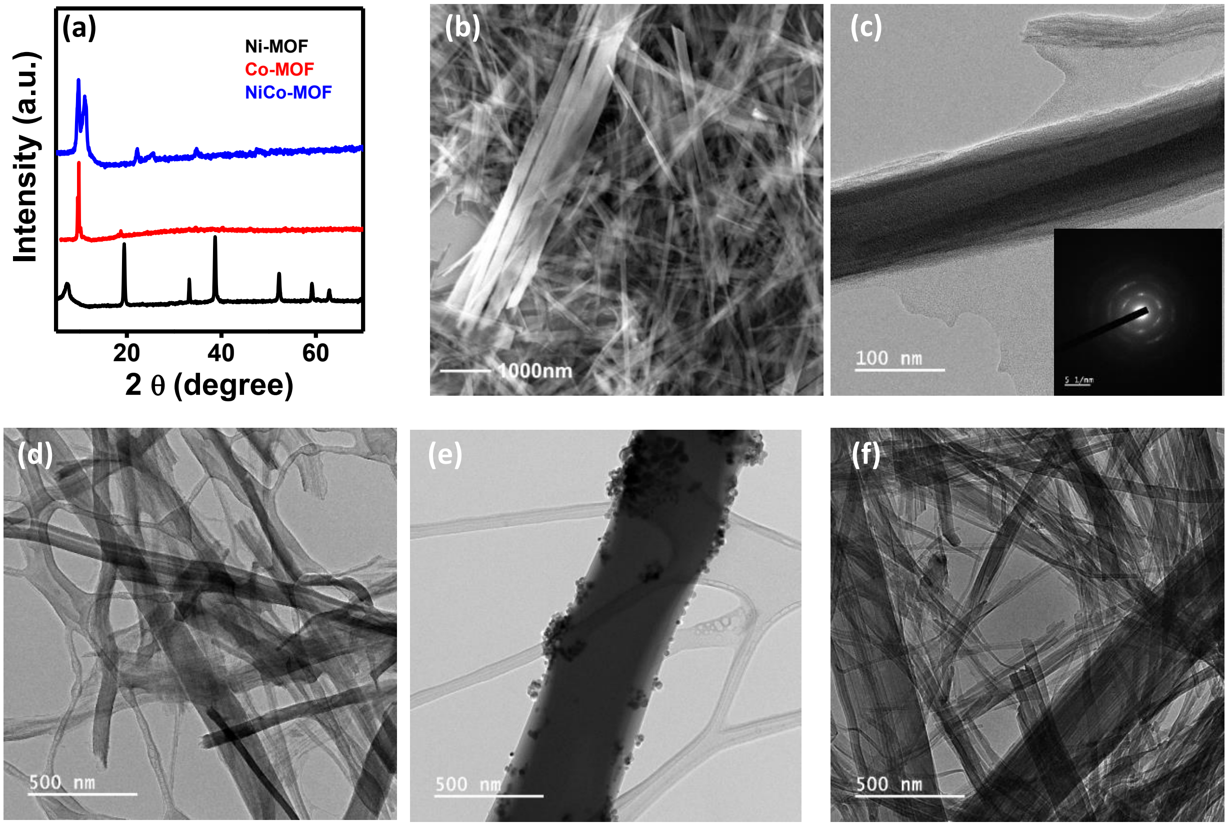

Figure S1. (a) p-XRD pattern of MOFs, (b) SEM image of NiCo-MOF, (c, d) SEAD and TEM images of NiCo-MOF, and (e, f) TEM images of Co-MOF, and Ni-MOF, respectively.

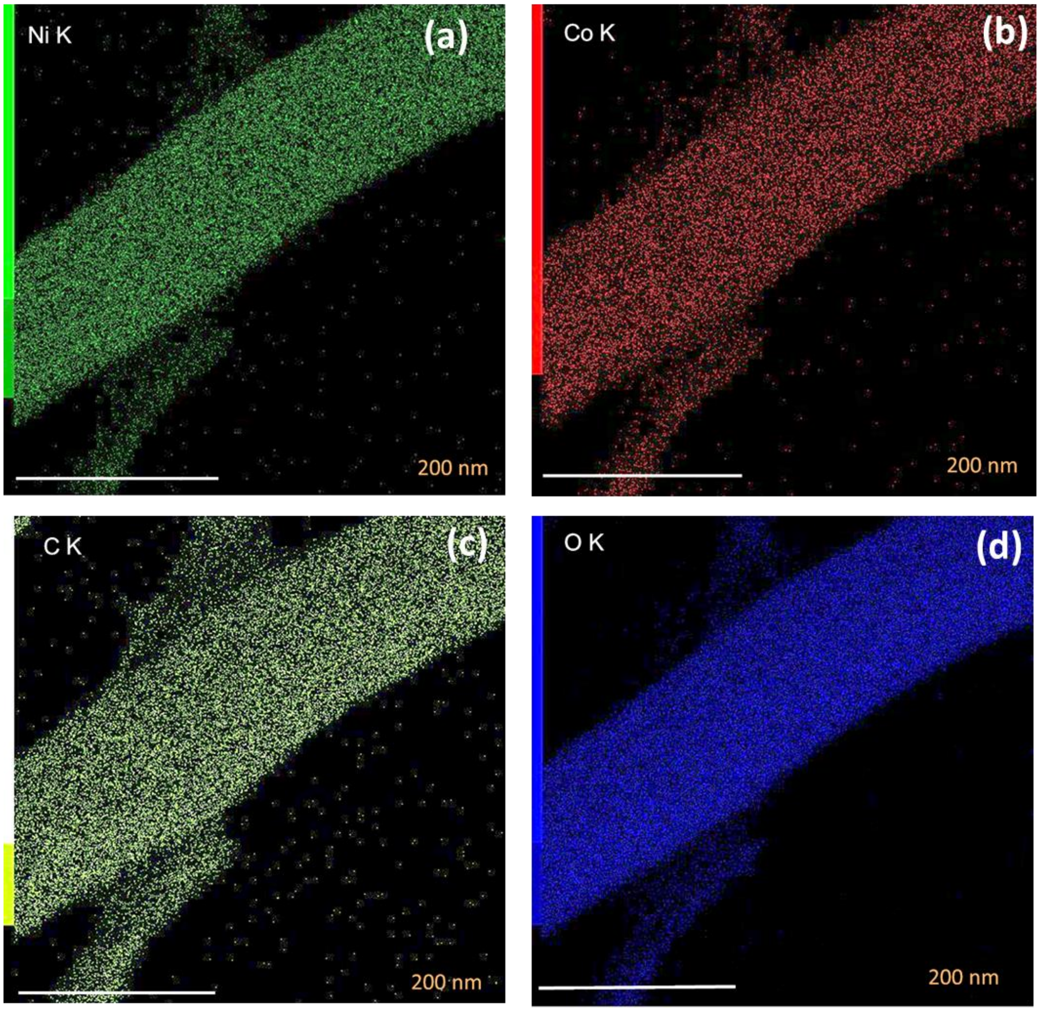

Figure S2. (a-d) Elemental mapping of NiCo-MOF.

Table S1. Elemental composition of MOFs determined by the TEM EDX spectra.

**Catalyst**	**Weight % of Ni**	**Weight % of Co**	**Weight % of O**	**Weight % of C**
NiCo-MOF	~ 16	~ 15	~ 33	~ 35
Co MOF	-	~ 46	~ 29	~ 24
Ni MOF	~ 28	-	~37	~ 34

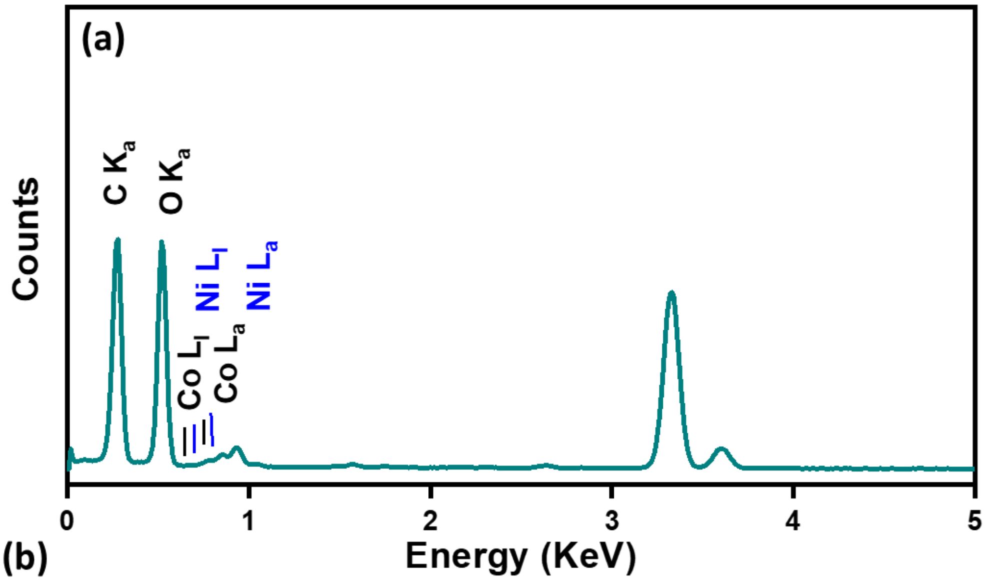

(b)

Element	Atomic % from XPS	Weight %
C	57.65	42.63%
O	36.41	35.85%
Co	2.55	9.25%
Ni	3.39	12.25%

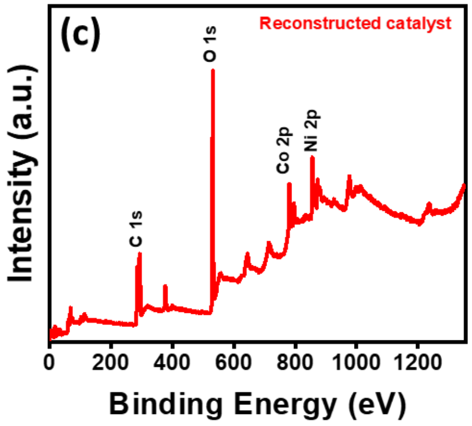

Figure S3. (a) EDS spectrum of reconstructed NiCo-MOF; (b) Elemental composition of reconstructed NiCo-MOF determined by the XPS spectra; (c) XPS survey scan of reconstructed NiCo-MOF.

Mechanism for OER:

Oxygen evolution reaction (OER) mechanism in alkaline medium

The reaction path involved for OER in an alkaline medium is given by the:

$4OH^- \leftrightarrow 2H_2O + O_2 + 4e^-$

The above reaction is followed by several steps involving four electron transfers.

$AS + OH^- \rightarrow AS\text{-}OH^* + e^-$; b ~ 60 mV

$AS\text{-}OH^* + OH^- \rightarrow AS\text{-}O^* + H_2O + e^-$; b ~ 40 mV

$AS\text{-}O^* + OH^- \rightarrow AS\text{-}OOH^* + e^-$; b ~ 24 mV

$AS\text{-}OOH^* + OH^- \rightarrow AS\text{-}OO^- + H_2O$

$AS\text{-}OO^- \rightarrow AS + O_2 + e^-$; b ~ 15 mV

Where AS denotes the active site on the surface of the catalyst.

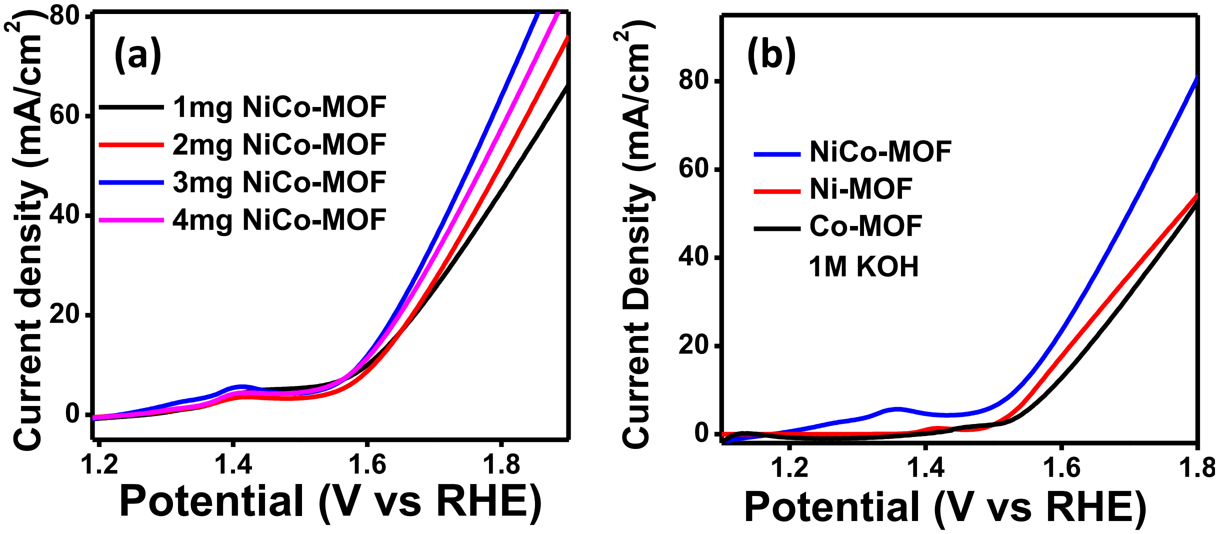

Figure S4. (a) OER LSV curves of NiCo-MOF at different loading; (b) Non-iR-corrected OER polarization curves of NiCo-MOF, Ni-MOF & Co- MOF.

Tafel plot:

For fitting the linear region of Tafel plots, the Tafel formula: $\eta = b \log (J) + a$, where η, J, b, and a are overpotential, current density, Tafel slope, and constant, respectively.

Where $b = 2.3RT/\alpha F$ (R - gas constant, α - symmetry coefficient, T - absolute temperature, F - faraday constant). During the electrochemical process, the faster electron transfer indicates the lower value of the Tafel slope (b).

Ethanol oxidation reaction (EOR) mechanism in alkaline medium

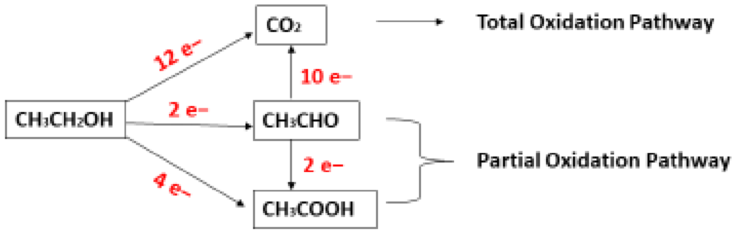

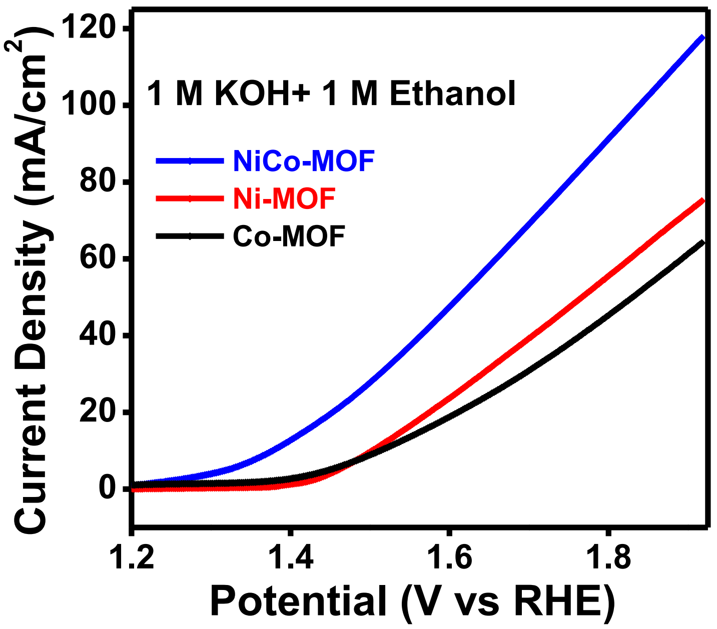

Figure S5. Non-iR-corrected EOR polarization curves of NiCo-MOF, Ni-MOF & Co MOF.

Table S2. The comparison of EOR activity parameters of NiCo-MOF with other reported catalysts in base media.

**Catalyst**	**Electrolyte**	**Potential (V vs. RHE) at 10 mA/cm^2**	**References**
$Ni_{0.75}Co_{0.25}Se_2$	1 M KOH + 1 M ethanol	**1.339**	*Chemical Engineering Journal,* 2022, 440, 135817.
**NiCo-MOF**	**1 M KOH + 1 M EtOH**	**1.33**	**This Work**
CoNi-PHNs	1 M KOH + 1 M EtOH	1.39	*Advanced Materials,* 31(28), 1900528. (2019)
Mo-Fe/Ni$(OH)_2$-3	1 M KOH + 1 M ethanol	1.352	*Journal of Colloid and Interface Science,* 680, 441-452. (2025)
$Ni_1Co_2P_x$ nanoparticles	1 M KOH + 1 M MeOH	1.38	*ACS Appl. Mater. Interfaces* 2020, 12, 34971-34979
CoS_2/CC	1 M KOH + 1 M EtOH	1.40	*Inorg. Chem. Front*. 2020, 7, 4498-4506
Co_3S_4-NSs/Ni-F	1 M KOH + 1 M EtOH	1.30	*ACS Appl. Mater. Interfaces* 2021, 13, 3, 4026–4033
$NiCo_2O_4$ nanosheets	1.0 M LiOH + 0.1 M glycerol	1.23	*Chinese Journal of Catalysis,* 57, 68-79. (2024)
NiOx/G	1 M KOH + 0.1 M Glycerol	1.364	*Dalton Trans*., 2024,53, 4237-4242
$NiCo_2O_4$/NF	1.0 M KOH + 0.1 M glycerol	1.13	*Advanced Functional Materials,* 34(3), 2306995.
Ni-Fe-P/NF	1 M KOH + 1 M EtOH	1.36	*Appl Surf Sci*, 561 (2021), Article 150080

Calculation of Electrochemical surface area (ECSA) calculation:

The electrochemical surface area of the reconstructed NiCo-MOF, Ni-MOF and Co-MOF was measured from the double-layer capacitance (C_{dl}). The amount of the electrical capacitance between the double layers can be measured from the CV curves at different scan rates by the equation

$$\nu \times C_{dl} = J_c$$

Where ν is the scan rate and J_c is the double-layer charging current. The slope obtained from the plot between the double layers charging current and scan rate is directly consider as the double layer capacitance (C_{dl}) of the catalyst. The ECSA value is directly proportional to double-layer capacitance by the following equation

$$ECSA = C_{dl} / C_{sp}$$

Where C_{sp} is the specific capacitance.

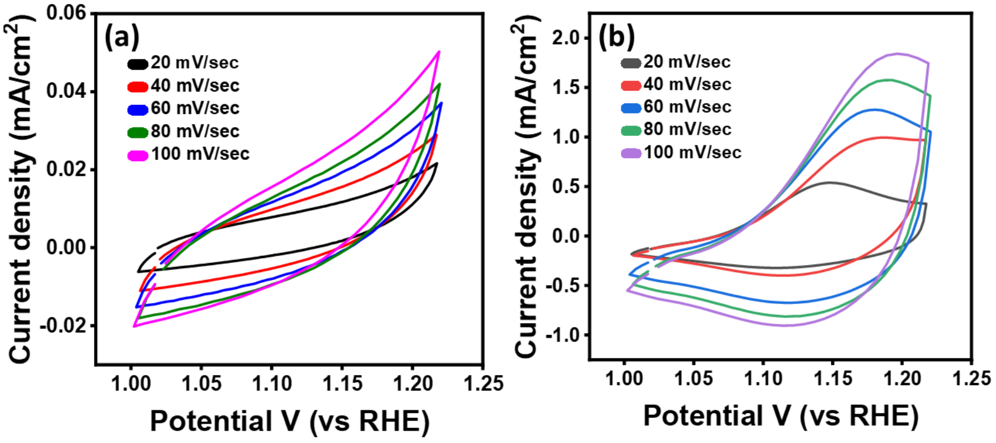

Figure S6. (a, b) CVs of Ni-MOF, and Co-MOF at different scan rates for OER.

Faradaic efficiency measurements:

To quantify the products formed in the ethanol oxidation process, a ^{1}H NMR with a water suppression technique (90% H_2O + 10% D_2O) was employed. The ethanol oxidation was carried out in a 30 mL electrolyte solution in the anodic part. For 1H-NMR analysis, 5mL of the reaction

solution and 3 μL of dimethylsulfoxide ($(CH_3)_2SO$) (as an internal standard) were mixed in a glass vial. Then, 450 μL of the solution and 50 μL of the D_2O mixture were used to collect standard NMR data of acetate. Then, the peak area of the internal standard (DMSO) was compared with the product (acetate) in MestReNova software for quantification of the product (acetate). The following equation carried out product quantification,

$$\frac{\mathrm{Np}}{\mathrm{Ns}} = \frac{\mathrm{Ap}}{\mathrm{np}} \Big/ \frac{\mathrm{As}}{\mathrm{ns}}$$

Np = no. of moles of product; Ns = no. of moles of standard; np = no. of protons corresponds to the chosen peak of product (which is 3 for acetate); ns = no. of protons corresponds to the chosen peak of standard (which is 6 for $(CH_3)_2SO$); Ap = area of product peak; As = area of standard peak. Therefore, the no. of moles of product (Np) can be calculated by finding the relative area of the product from the software and the corresponding no. of moles of standard. The relative area can be found from the NMR spectra.

$$\text{The Faradic efficiency (\%)} = \frac{\text{number of moles of product formed experimentally for a certain time}}{\text{Theoritically calculated moles of product for the same time}}$$

The theoretical amount of product was calculated from the accumulated charge during galvanostatic electrolysis by assuming 100% faradic efficiency.

$$\text{Theoretical amount (n in mole) of product (acetic acid)} = \frac{Q}{\mathrm{n} * \mathrm{F}} = \frac{\mathrm{I} * \mathrm{t}}{\mathrm{n} * \mathrm{F}}$$

Where I is the current in Amp, t is time in sec, n is the number of electrons, which is 4 for EOR (acetic acid), and F is the Faraday constant (96485.3 C mol^{-1}).

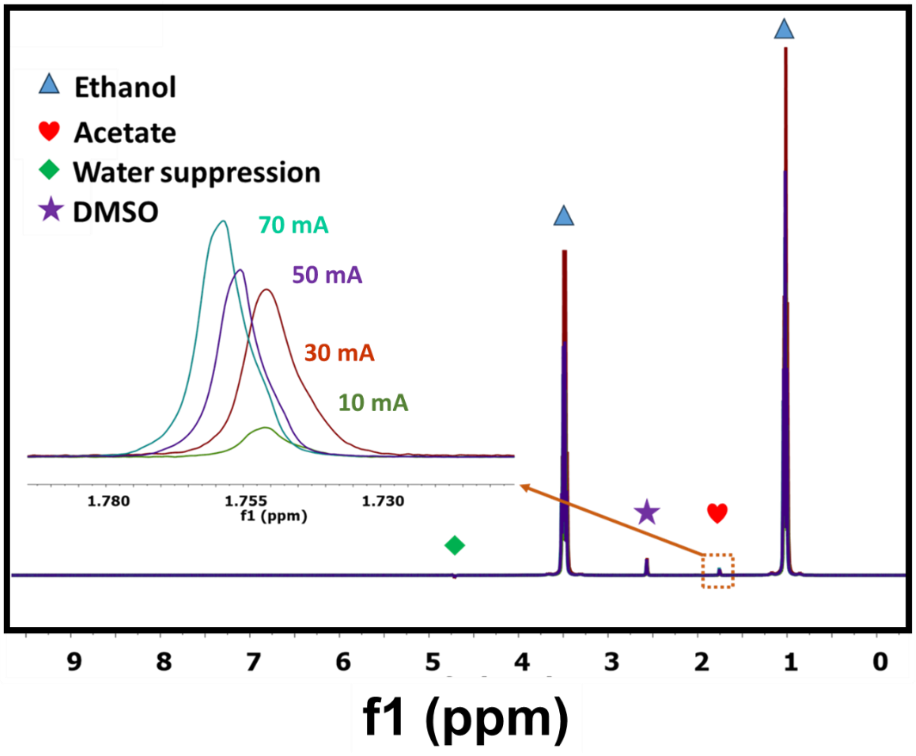

Figure S7: NMR plot of the reaction mixture performed at different current densities of the reconstructed NiCo-MOF.

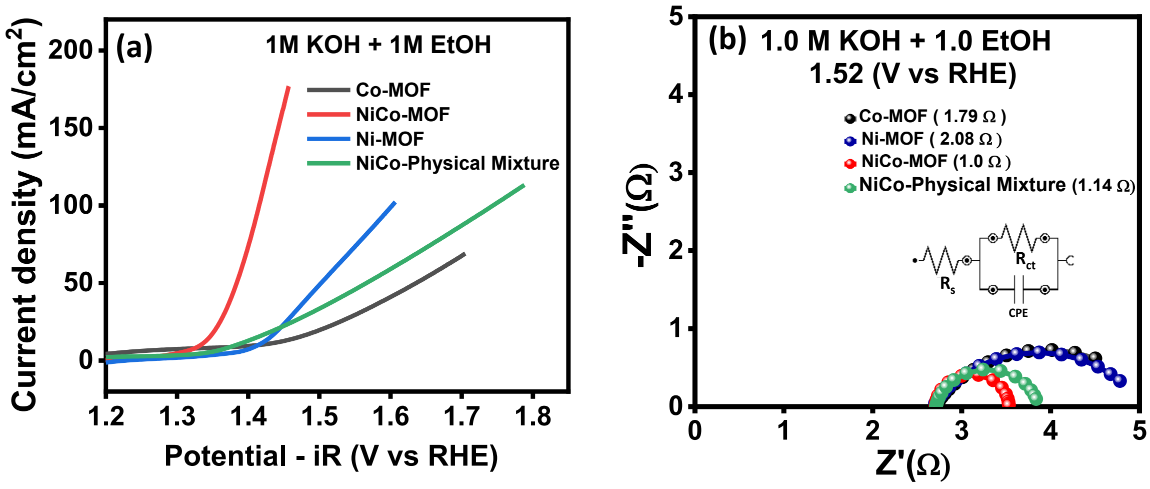

Figure S8: (a), (b) are LSV and Nyquist plot for NiCo-MOF, Co-MOF, Ni-MOF and physical mixture respectively.

Electrode preparation of Overall water and Ethanol electrolysis:

The overall water splitting was performed in 1.0 M KOH with 1.0 cm^2 carbon cloth as a working electrode. Before doing the electrochemical study, the carbon cloth is washed sequentially in water, ethanol, 5% HCl solution, and then again in water and ethanol each for 2 min. The anode electrode was prepared by drop casting the aqueous suspension of NiCo-MOF composite on the cleaned carbon cloth with a MOF loading of 3 mg cm^{-2} and dried at room temperature. The cathode electrode was prepared by drop casting the aqueous suspension of commercial Pt/C composite on the cleaned carbon cloth with a catalyst loading of ~ 1.5 mg cm^{-2} and dried at room temperature.

Overall water-splitting mechanism:

Generally, bifunctional electrocatalysts of HER-OER are typically used as the cathode and anode to speed up the overall water-splitting reaction. O_2 evolution and H_2 generation occur at the anode and cathode, respectively, when an external potential is applied between the two electrodes. Irrespective of the reaction media, the ideal thermodynamic voltage for water splitting is 1.23 V (25 °C and 1 atm). The water-splitting mechanism can be described as:

The total reaction involved - $2H_2O \rightarrow 2H_2 + O_2$

In alkaline solutions: Cathode: $4H_2O + 4e^- \rightarrow 2H_2 + 4OH^-$

Anode: $4OH^- \rightarrow 2H_2O + O_2 + 4e^-$.

Overall ethanol electrolysis mechanism:

In Overall Ethanol Electrolysis, the two half-cell reactions are HER at cathode and EOR at anode. H_2 generation takes place at cathode. In this case, both electrodes were made by fabricating the material on the carbon cloth covering the 1 × 1 cm^2 area. A customized H-cell was used for the experiments with a 15 mL electrolyte solution containing 0.1 M ethanol in 1 M KOH for the anode

chamber, but for the cathode chamber, only 15 mL of 1 M KOH solution was used, and both compartments were separated by a "Nafion 117" membrane to avoid the reduction of acetate at the cathode. For products at the anode, there are two possible pathways mainly giving C_1 and C_2 products. Going via C_1 pathway the complete oxidation of ethanol to CO_2 is possible by delivering 12 electrons. For C_2 pathway there is the oxidation of ethanol to acetate take place by delivering 4 electrons or to acetaldehyde by delivering 2 electrons without breaking the C-C bond. The overall ethanol electrolysis mechanism can be described as:

The total reaction involved -

$$CH_3CH_2OH + xH_2O \rightarrow 6H_2 + 2\ CO_2 \text{ (Considering complete oxidation)}$$

In alkaline solutions: Anode: $CH_3CH_2OH + xH_2O \rightarrow$ C derived products + $yH^+ + Ye^-$

Cathode: $2H^+ + 2e^- \rightarrow H_2$
